# Microfluidics - The State-of-the-Art Technology for Pharmaceutical Application

**DOI:** 10.34172/apb.2022.073

**Published:** 2021-09-29

**Authors:** Anisha Verma, Sayani Bhattacharyya

**Affiliations:** Krupanidhi College of Pharmacy, Bengaluru, Karnataka 560035, India.

**Keywords:** Microfluidics, Drug discovery, Product development, Nanoparticles

## Abstract

Microfluidics (MF) is the science dealing with the behavior, precise control, and manipulation of fluids as well as particles on the scale of tens to hundreds of micrometers. It is also utilized for chemical and biological applications, usually called micro–total analysis systems (µTAS) or lab-on-a-chip (LOC). MF is a fascinating and capable technology with various superior benefits compared to conventional macro-scale platforms, such as the lesser requirement of sample and reagent volumes, higher sensitivity, low cost, portability, faster processing of samples and potential to be automated and highly integrated to reduce human errors. The concept of transformation of meso to nanoliters using MF technology has shown its potential in the healthcare system for early diagnosis, and personalized medicine. The integrated multifunctional system with parallelization provides a better and faster process control. Minimization of the consumption of fluid makes the technology safer in every aspect of the development process, analysis, and storage. The impressive improvement in patient care and monitoring has led to the commercial motivation of the pharmaceutical industry to develop new drugs and modify existing products with better efficacy and safety in a cost-effective manner using MF technologies. Hence, the present review briefs on the applications of MF technology in the key issues of the drug discovery process, overcoming the limitations of development of analytical procedures and prosperous pharmaceutical manufacturing for novel controlled and targeted release dosage forms to fabricate quality products.

## Introduction

Microfluidics (MF) is the science and engineering of processing 100 nL to 10µL of fluid volume (e.g., reagents and samples) and manipulating them in microchannels having a minimum of one dimension (such as channel diameter, depth, or width) with 10-100 µm length scale.^1^ Microfabrication has improved the electronic and biochemical industry by the introduction of micro-electro-mechanical-systems (MEMS) and micro-total-analysis-systems (µTAS) respectively.^2,3^ Later, lab-on-a-chip (LOC) technology, a miniaturized model, was introduced as a subdivision of MEMS and is widely used for analytical and non-analytical purposes. It utilizes the science of MF and converts a typical bench-top laboratory on a small chip. Technically complex laboratory procedures can be performed on a chip in an automated and integrated manner. Different sorts of components such as valves, heaters, motors, and other functional units have been miniaturized using MF technology along with detection and sensory systems, with the inclusion of electric, magnetic and optical detection.^4^

The reduction from macro to micro length scale results in unique and important non-intuitive phenomena at the microscale level. Fluid flow can be laminar or turbulent, and depends on the relative contribution of viscous and inertial forces on fluid flowing through a channel, and described by Reynolds number (*Re*). In MF systems, *Re* is below 100 or below unity, which resembles a laminar fluid flow dominated by viscous forces. This property of the flow of fluid leads to the selection of the design of the MF devices based on laminar fluid diffusion interfaces.^5^ The design for sample introduction or extraction through an MF system is associated with two methods- induction of electroosmotic flow in the fabricated materials of MF channels and pressure-driven flow using a displacement pump. Hence optimization of the fluid flow is a critical step to achieve a successful MF system.^6^

Digital MF (DMF) is a corresponding and well-defined technology having wide applications in pharmaceuticals, chemistry, and biology.^7^ In this technique, individual droplets of reagents or samples after being dispensed from reservoirs are split, combined, and then mixed with high precision on an open surface^8^ and a series of electrical potentials is applied to an array of electrodes.^9^ Some benefits of DMF are: (i) easy manipulation of samples and reagents with no need for tubes, pumps, and microvalves, (ii) compatibility with organic and aqueous solvents, (iii) can handle vast ranges of volume (nL-mL) and (iv) direct control over distinct phases.

Sista et al^10^ extracted DNA from whole blood samples by using magnetic beads, and analysis was done by polymerase chain reaction (PCR) and immunoassays by employing a DMF technique which was developed by their team. Mousa et al^11^ established a DMF technique for the procession of 1 mg sample of breast tissue homogenate and a 1 µL sample of serum and blood for quantification of steroid hormones.

**Figure 1:**
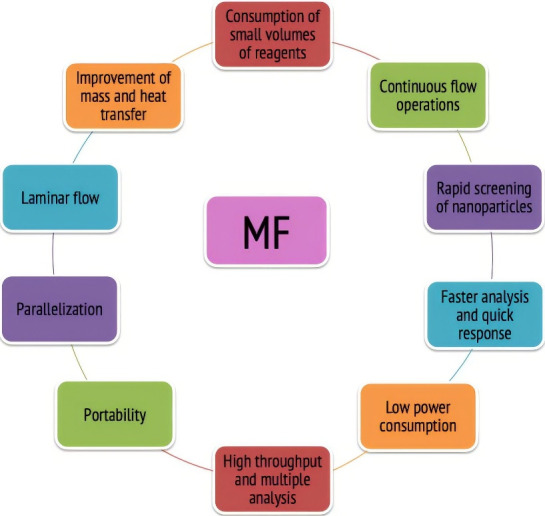
Benefits of MF technology

There are numerous unremitting benefits of MF technology, a few of them are mentioned below in Figure 1.^12,13^

It is desirable in the upcoming era of science due to its size effect and hence portability, reduced sample or reagents consumption, and shorter time for assay. It is also widely used in the pharmaceutical field from the early stages of drug discovery, screening and to the final stages of controlled and targeted delivery.^14^ This novel technology also supports a system called “organ-on-a-chip”, which mimics intricate cell-cell interactions and cell-microenvironment interactions, that are found in biological organs and tissues (e.g., kidney, lungs, liver, and heart).^15,16^ Hence, this review encompasses the novel techniques of MF in various aspects of drug discovery and pharmaceutical operations.

## Materials used for fabrication of MF devices

Vast ranges of different materials have been used for MF devices fabrication. A common fabrication method for MF devices or chips is soft lithography, which includes the following techniques: wet-etching, hot embossing, and micro-contact printing.^17^

Traditionally, the dominant preferences for MF fabrication were silicon and glass due to their advanced micromachining procedure and easy availability.^18,19^ But due to their disadvantages such as expensive processing costs, lack of self-sealing ability and brittleness lead to the development of new flexible materials which could bend and stretch, and deform under mechanical loads to fabricate newer generations of MF devices. Such materials include parylene, PI (polyimide), PET (polyethylene terephthalate), OSTE (off-stoichiometry thiol-ene), PDMS (poly(dimethylsiloxane)) and Ecoflex.^20^ Some common forms of polymers used for MF chips are beads, membranes, fibers, and porous polymer monoliths.^21^

PDMS is a siloxane elastomer and the most broadly used material for the fabrication of MF devices.^22^ Parylene is a thermoplastic polymer that can be formed as an ultra-thin film, leading to a reduction in bending stiffness.^23^ Parylene-based MF devices have various biomedical applications, such as, implantable 3D probes for drug delivery,^24^ injection tools, and neural probes.^25-27^ PET is also a thermoplastic polymer whose geometrical properties (e.g., thickness and controlling crystalline and amorphous contents) when adjusted yield highly flexible PET substrates. Lin et al^28^ employed the technique of laser cutting to fabricate 3D MF devices, which were flexible and wearable, by employing transparent PET films by developing a low cost and scalable method. PI is a biocompatible polymer, is used for medical applications. It has been utilized in point-of-care systems and biosensors.^29^ PI diaphragms have been employed for manufacturing micropumps, owing to their good sealing properties.^30^ OSTE is a UV-curable polymer, that comprises excess of unreacted allyl or thiol groups, bonded together under UV exposure. Chen et al^31^ fabricated an MF device with OSTE polymers for electrochemical detection.

## Applications of MF in drug discovery

 MF is a useful technique for drug discovery and development, as it can reduce the processing costs and time and make tasks easier.

For drug discovery, robotics or automatic analysis systems are utilized, like the high-throughput screening method (HTS). But these methods require longer processing time and are costly. MF can be used to limit such barriers.^32^ It can be useful for avoiding several such limitations in each step of the drug discovery and development process, as mentioned below.

###  Selection of target and validation

####  Protein analysis in a single cell

Single-cell analysis is useful for the investigation of cell-to-cell heterogeneity in a big population. But there are several challenges for probe protein information at single-cell resolution, which can include interactions, dynamics, and quantity. These challenges arise due to the size of the cells, their complex nature, huge range of protein concentration, and absence of genome-wide amplification techniques. MF devices can be used to detect minute amounts of proteins for single-cell content quantification. These MF devices can cause manipulation, lysis, separation, and quantification of protein contents of single-cell by utilizing the process of single-molecule fluorescence counting. There are various aspects of MF techniques by which single-cell protein analysis can be carried out, including MF electrophoresis, MF probe, MF cytometry, MF array (including microchambers, microwells, static droplet array MF, valve-based MF), and MF based mass spectrometry.^33^ An MF device was utilized for the measurement of many epitope-tagged human β_2 _adrenergic receptors.^34^ In another study, MF devices were integrated with an electrophoretic separation procedure, and analysis of amino acids was carried out for the lysed contents of single-cell.^35^

###  Protein separation and crystallization

Conventionally, 2D polyacrylamide gel electrophoresis (2D PAGE) is used for protein separation, but it has disadvantages like low sensitivity, low-performance efficiency, and requirement of a larger number of samples.^36^ Therefore, MF-based approaches are used for protein separation, like size-based separation and capillary electrophoresis. An MF approach with capillary gel electrophoresis and isoelectric focusing has been developed.^37^ In a recent study, a MF chip was used to separate the protein molecules to develop a 2D fingerprint of a heterogeneous mixture of proteins.^38^

Determination of macromolecular structure by protein crystallization is usually the rate-determining step.^39^ MF systems are useful in this case. For instance, an MF platform for protein crystallization was developed which was integrated with 144 parallel reactions and 480 on-chip valves. Protein crystallization was carried out by free interface diffusion.^40^

###  Ligand binding

In the path of drug discovery process selection of ligand and binding of small molecules to macromolecules is a critical step. MF can be utilized in ligand binding for improvement of sensitivity, throughput and reduce interaction times. Assessment of the degree of interactions at the molecular level is a challenge in the traditional high throughput screening. In such a case, a high throughput MF device has been used for the characterization of the binding energy of DNA by utilizing four eukaryotic transcription factors. This device can be utilized for testing the binding of each transcription factor to DNA and also for the prediction of their *in vivo* functionalities.^41^ MF cantilevers or magnetic nanosensors can be utilized in the quantification of particular ligand binding interactions.^32^ Magnetic nanosensors in MF systems have been used as affinity ligands in HTS to detect all molecular interactions in disperse magnetic particles, that can be further utilized as affinity ligands for HTS applications in MF systems.^42^ Burg et al^43^ developed an MF cantilever chip for weighing and analysis of biomolecules, single nanoparticles (NPs) in fluid and single cells.

###  Hit molecule identification and optimization

The key processes in the drug discovery process are identifying hits and their optimization into leads.^44^ The probable pool size of drug candidates for this process is huge and of the order of 10^63^. Hence utilizing conventional macroscale methods for the generation and optimization of drug candidates can be an exhaustive process. Microscale technologies are more useful in generating drug candidates owing to their lesser reagent volume requirement and short reaction times. They can also be utilized for the synthesis of chemical libraries and hence enhance the chances of discovering new chemical entities.^32^

###  Lead generation

Microreactors can be used to generate lead compounds to carry out a vast number of chemical syntheses, discussed widely in the manufacturing section. For instance, a glass microreactor was used to prepare peptide derivatives from α-amino acids within 20 minutes.^45^

This technology is also useful to generate synthetic genes for biological applications like DNA synthesis. For example, MF devices have been used for mRNA isolation and cDNA synthesis.^46^ An MF device has been fabricated for oligonucleotides synthesis and purification. The device was programmable and enabled the rapid generation of a vast quantity of oligonucleotides.^47^

Natural drug candidates can also be assessed using MF technology, as their popularity is rising every day and have an annual growth rate of 5% to 15%.^48^ MF can be utilized for the identification of specific natural drug candidates responsible for the therapeutic action, from a wide array of constituents present in the parent substance. For instance, for the screening of natural medicines, multi-electrode microchips were developed. These microchips were sensitive and selective enough for the screening of complex mixtures of neuroactive molecules. They were also employed for the parametric study of phytochemical extracts from several plants.^49^

###  High-throughput screening

HTS is important for the identification of hit compounds. It is a major tool for screening the properties of new chemical entities. Conventionally, HTS systems use multiple-well plates, but due to difficulty in dispensing nanolitre volumes of liquids into the wells, their miniaturization is difficult. Therefore, in such cases, MF can be useful. MF technologies like gradient-generation, microwell arrays, multiplexed systems and, plug-based techniques can be employed for the achievement of accuracy in the screening process.^32^ For instance, mRNA and DNA purification was performed by using an integrated MF device with on-chip valves using multiplexed systems.^50^ Microwell arrays method was employed to analyze the response of various cell types, such as fibroblasts and hepatocytes, to various compounds. Microwells for cell seeding were integrated within MF channels to analyze the cell responses.^51^

In a recent study, it was revealed that a screening of six potential antibiotics was hastened from thousands of small molecules, which were emulsified with bacterial cells and barcoding dyes into 1nL nanodroplets in a 4 million well MF device.^52^

###  Preclinical studies

####  In vitro studies

By using MF devices, the screening process becomes easier and cheaper than conventional *in vitro* and *in vivo* methods. MF systems comprising of a network of interconnected chambers can be utilized to mimic the actions of tissues, to recreate the cell-cell interactions and pharmacokinetic and pharmacological interactions between tissues and organs, which can be used to study toxicology. In such a system, each compartment can be made to represent a specific organ or cells like fat cells or lungs to mimic their functions in the body.^53^ Such cell-based MF systems can also be used to study the collaborative effect of combinatorial drugs.^54^ In recent study perfusion of anticancer drug etoposide in HeLa cell lines was studied using MF devices integrated with 96 well plates. This led to the removal of the constant pumping requirement of media throughout the period of experimentation, and a scale-up was possible by integrating the MF device with 384 well plates.^55^

###  In vivo studies

*In vivo* studies are done to test the pharmacological actions of drug candidates. Blood samples have to be obtained from animals, most of the time blood withdrawal from the small animals will be challenging. In such cases, MF systems can be used.^56^

Extraction of drug compounds for animal tissues analysis can be done by MF systems. For instance, antivenom antibody from soluble proteins was separated by MF device, and the yield was 100% better than the yield obtained from centrifugation due to reduced losses of materials.^57^

In cancer, circulating tumor cells, tumor cell-endothelial cell interactions, and chemical mediator exchange between them are of great interest that can be studied by MF platforms. Zheng et al^58^ monitored chemical mediator exchange between tumor cell-endothelial cells by the use of a pressure-driven MF system, eliminating any actual physical contact interference.

###  Clinical trials

####  Human blood sampling and processing

Human blood sampling needs to be designed in such a way that the trauma for the patient is minimal and also to provide maximum assistance to doctors. These MF devices have the benefit of alleviated trauma for patients and decreased costs. An MF disk with a plasma extractor (utilizes 500 nL volume) can be utilized to purify plasma, measure purified plasma and then convey the purified plasma into the detection chamber for analysis.^59^

To allow minimally invasive and pain-free sampling of blood, a titanium microneedle with the size equivalent to that of a female mosquito’s labium, i.e., 60 µm outer diameter and 25 µm inner diameter, was developed. The design of this microneedle was based on the mosquito’s mechanism of blood extraction. This device can also be an alternative for blood sampling by patients.^60^

###  MF for toxicity study on in-vitro cell lines

####  Toxicity induced by drug on tissue and cell culture

Before the market release of a drug product, its toxicity studies have to be performed to ensure that the product is safe for human use. MF techniques can be utilized here to lessen the time and amount of chemicals consumed.^2^

###  Hepatotoxicity

A liver-on-a-chip was developed to mimic natural sinusoids using MF technology. It consists of a channel for nutrient and drug flow and a cell compartment. It also has a permeable endothelial-like barrier of sinusoidal shape to resist high shear stress and pack hepatocytes in the cellular area. Rat and human liver cells were added to the cell area of the chip and screened for cell viability for a period of 7 days. Results revealed that both cell lines were found to maintain their viability.^61^ In another study, an MF device with primary human hepatocytes trapped in microholes was fabricated for demonstration of a hepatotoxicity assay system. MF hepatotoxicity assay of various drugs affecting liver function like acetaminophen, diclofenac, verapamil, and benzopyrene was carried out. A mathematical hepatotoxicity model was also developed based on the time-dependent cell death profiles that were measured by the MF device.^62^

###  Cardiotoxicity

Cardiovascular toxicity is the main cause for the elimination of a drug from getting FDA approved. Hence the demand for new drugs with no cardiotoxicity is high. In a recent study, cell-derived tissue chips, human induced pluripotent stem were integrated into a 3D-MF system for pre-clinical drug testing. A study with doxorubicin and oxaliplatin was found to be successful in estimating the effect on cardiotoxicity, and determination of IC_50_. The findings were found to be consistent withthe* in vivo* studies.^63^

In another study, beating *in vivo*-like human cardiac bodies were employed in an MF device, which exhibited similar structural and functional properties as that of human myocardium. For automated monitoring of the beating frequency of each cardiac body on an MF device, a video-imaging technique was employed. Beating frequency data for 6 hours were compared to literature data and resulted in a novel non-invasive technique for detecting cardiotoxicity.^64^

###  Cytotoxicity

A 3D cytotoxicity assay method was developed using high-throughput MF 3D Cytotoxicity Assay for Cancer Immunotherapy (CACI-IMPACT), to detect killing properties of cytotoxic lymphocytes in a 3D microenvironment. This was done via spatiotemporal analysis of cancer cells and lymphocytes, which were embedded in the 3D extracellular matrix (ECM). It was found that 3D ECM lessened cytotoxic lymphocytes migration and hence their access to cancer cells.^65^

Phenotype-based cytotoxicity assessment can also be performed utilizing the MF technique. In a study, zebrafish embryo was selected as target model to carry out the toxicity of cisplatin, doxorubicin, vitamin C, and 5-fluorouracil (5-FU) on phenotype characteristics using an MF system with serpentine-shaped microchannels. The process was successfully employed for estimating the drug toxicities at the developmental stages.^66^

Different MF techniques used for cytotoxicity assessment and enzymatic assays^67^ are listed in Tables 1 and 2.

**Table 1:** MF techniques for Cytotoxicity assessment

**MF technology**	**Applications**	**Reference**
3D hydrogel matrices	Study of biological activities of molecules in a matrix of multicell biomimetic tissue or organ culture, example Matrigel®	68
Dielectrophoretic MF	Production of the homogeneous cell population for regenerative medicines and tissue engineering	69
Digital MF	An alternative technology to lab-on-a-chip systems to Conduct biological processes for cytotoxicity assessment	70

**Table 2:** Enzyme inhibition assays using droplet MF

**Droplet MF**	**Application**	**Advantages**	**Reference**
Static droplet array	Simultaneous study on enzyme kinetics and its inhibition with a low volume of samples	Analyzed more numbers of partitions ( > 1 million) Enhanced multiplexing and sensitivity Capability for downstream sorting of the droplets	71
Mobile droplet array	Perform assays of time-dependent steady-state enzyme kinetics and inhibition	Precise control over poly-dispersity and size of droplets	72

## Pharmaceutical applications

###  Manufacturing

Automated and miniaturized microreactors employing the MF technique can be utilized for the synthesis of chemicals due to their high speed and efficiency rate.^73^ In a microreactor, an MF segment has a minimum unit which can be used to improvise several reactions and unit operations in micro space. Profound uses of microreactors are seen in HTS in microanalytical chemistry, reactions kinetics studies and their mechanism, and biological analysis of cells and proteins. The advantages of microreactors include their easy application and precisely controlled contact time, size, and shape of the interface between fluids and higher mass and heat transfer rates. Microreactors can be of two types: chip type and microcapillary microreactors. Chip type microreactors have several advantages over capillary microreactors, such as integration of various processes into a single reaction device and easy control of MF.^74^ These microreactors can be fabricated from various materials like glass, silicon, metals, quartz, etc. by methods like injection molding, photolithography, powder blasting, hot embossing, and laser micro forming.^75^ They can be conveniently scaled up for large-scale manufacturing. Reaction conditions can be optimized due to the ability of MF to perform high throughput experiments. A continuous flow MF-based microreactor has been utilized to study glycosylation in organic transformation.^76^ Shah et al^77^ utilized the capillary MF technique for the production of thermosensitive monodisperse poly (N-isopropyl acrylamide) gel particles which were in the size range of 10-1000 µm. Their technique had great control over the inner morphology and outer dimensions of the particles. This technique is also useful for generating higher-order supra-particles, which can be done by the direct assembly of colloidal particles in droplets.

###  Product lifecycle management

The drug’s lifecycle after its launch should be extended, modified, and improved by employing a new dosing regimen, latest therapeutic indications, target patient populations, and by manufacturing modified formulations. Appropriate excipients can be used to improve dosage forms. Elaborative research on Cremophor EL (a mixture of hydrogenated castor oils)-free paclitaxel formulation (TaxolÒ) was carried out through this technology to identify the best combinations of excipients and also to minimize the toxicity associated with it. A full factorial combination of various combinations of paclitaxel with different excipients or excipient combinations at three different concentrations was screened using the MF technique. Out of 9880 combinations tested, only 19 were hit combinations, which were optimized to obtain the final formulation. Upon testing in rats, the optimized formulation was found to be well tolerated at low and high doses while on the contrary, TaxolÒ killed all the rats at high doses. The optimized formulation also exhibited slower elimination than TaxolÒ.^78^

###  MF for local drug delivery

Local drug delivery systems have the main goal of supplying a therapeutic level of drug to the desired physical site for a prolonged period and avoiding the delivery of drugs to non-target tissues to prevent systemic toxicities.^79^ The various challenges in the development of a local drug delivery system include drug-targeting inefficiency and the inability of long-term delivery, especially for short half-life therapeutic agents. These two issues can be resolved by the use of sustained-release and controlled release drug delivery systems, which can maintain constant drug plasma concentration within the therapeutic window.^80^

 MF implants offer advantages of sustained or controlled release of drug with precise control overflow for local drug delivery and can assure targeting of drug at the site of action.

###  MF for delivery to the skin

A device for MF transdermal drug delivery was fabricated in the form of hollow out-of-wafer-plane silicon microneedles. The needle arrays offered reduced resistance to liquid flows and a bigger surface area between tissue and fluid. They were suited for drug or vaccine delivery.^81^ Lukács et al^82^ fabricated a skin-on-a-chip device for transdermal delivery of drugs and their monitoring which can be used for dermal pharmacodynamics and pharmacokinetic studies. It had advantages of being low cost, low volumes of sample, and providing rapid and reproducible results.

###  MF for delivery to the inner ear

Drug delivery to the inner ear is challenging because of its physiology, complexity, and inaccessibility. Hence MF systems can be used for delivery to the inner ear.^83^ A fully implantable reciprocating inner ear MF drug delivery device was developed which could enable timed and sequenced drug delivery into the cochlea’s perilymph. Flow characteristics were tested in guinea pigs by using 6,7-dinitroquinoxaline-2,3-dione and were capable to alter auditory nerve responses. The device was found to be safe and effective in terms of drug delivery.^84^ Kim et al^85^ developed an advanced and wearable drug delivery device. Enhancements were made to the system by embodiment of planar micropump for generation of reciprocating flow and a novel drug reservoir to achieve constant predetermined rate of delivery of drug. The *in vitro* response and *in vivo* response were tested and found to be effective in long-term therapeutic efficacy in humans.

###  MF for ocular drug delivery

Conventional ocular drug delivery methods have limitations due to anatomical and physiological barriers and poor patient compliance.^86^ A major factor among anatomical barriers is the precorneal factors such as tearing, blinking, and low permeability of drug through the corneal membrane.^87^ These can be overcome by employing MF systems for prolonging drug release time. The newly developed MF systems also allow refilling micropumps so that the reservoir can be refilled once the drug is depleted, and can be used to administer different drugs from the same device.^88^

###  MF for delivery to the brain

The blood-brain barrier is a major challenge for drug delivery to the brain. All drugs used to treat neurological disorders ultimately affect tissues throughout the body. To avoid such limitations, MF systems can be utilized.^80^ Flexible polymer MF device has been developed for long-term drug delivery via implants, having a rigid scaffold with poly(lactic-co-glycolic acid). This system is small in size and has low rigidity. The scaffold helps to support the flexible polymer device but it breaks up in the tissue to release fluid.^27^

###  MF in cancer therapy

NPs are synthesized by MF technology using the bottom-up approach as mixing in microchannels requires lesser time and small NP are obtained with narrow size distribution. Surface functionality can be added to NP using MF.^89^ Some targeted drug delivery systems for the treatment of cancer employing MF technology are listed in Table 3.

**Table 3:** Examples for MF in cancer therapy

**Carrier**	**Drug**	**Excipient**	**Method**	**References**
Polymeric NP	Cisplatin, docetaxel	Polylactide (PLA)	Nanoprecipitation	^90^
Lipid-based NP	Bcl-2 antisense deoxyoligonucleotide (ODN)	Protamine, lipids (3β-[N-(N′, N′-dimethylamino- ethane)-carbamoyl] -Chol:egg and Phosphatidylcholine:1,2-distearoyl-sn-glycero-3-phosphoethanolamine-N-[amino(polyethylene glycol))	Modified ethanol dilution	^91^
Nanoemulsion	Tamoxifen	Soybean oil, Polysorbate 80	Ethanol injection	^92^
Lipospheres	Doxorubicin	PDMS	Photolithography	^93^
Microcapsules	Glucose-responsive 3-aminophenyl boronic acid (AAPBA) moiety	Poly (N-isopropyl acrylamide), acrylic acid	Free radical polymerization	^94^

###  MF in the fabrication of nanoparticles for controlled and targeted drug delivery

NPs are widely used in the pharmaceutical field due to their several advantages, such as small size and vast surface area, modifiable surface chemistry, high drug loading capacity, and high surface-to-volume ratio.^95^ At the same time, it also has some disadvantages, which can limit its future evolution. They have high batch-to-batch variation, poor control of conventional batch method NP preparation, and complications of NP-drug moiety.^96^ MF technology is being used in NP preparation using the nanoprecipitation method of preparation in microscale MF channels along with the continuous flow.^97^ This technology has the benefits of encapsulating different types of drugs, imaging or targeting moieties, and a 100% theoretical encapsulation.^98^ Some examples of different types of NPs fabricated by MF have been listed in Table 4.

**Table 4:** MF in the fabrication of NPs

**Process**	**Type of NP**	**Advantages**	**Reference**
MF continuous flow	Crystalline drug NPs	Production of ultrafine homogeneous NPs Uniform encapsulation	^99^
Microfluidic nebulator in supersonic spray dryer	Amorphous drug NPs	Solubility enhancement Less tendency to re-crystallize Improved stability for hydrophobic molecules	^100,101^
MF channels with tunable hydrodynamic flow	Polymeric NPs	Classic hold on composition and processing parameters Good control on shape, size and encapsulation efficiency	^102,103^
MF device with a multi-inlet micromixer	Targeted NPs	Enables employment of targeting ligands at different ligand densities and production of homogeneous tunable NPs	^104,105^
Droplet MFs	NPs in microsphere	Very high encapsulation efficiency with the use of nanoliters of immiscible solutions for production of homogeneous nanotube loaded microparticles for Dual drug delivery and sustained action	^106,107^

###  Droplet-based MF techniques

This technique is based on microdroplets production having characteristics such as precise volumes and dimensions, limited cross-contamination, and limited dispersion, which are ideal for the development of complex particles for smart delivery systems. A single emulsion can be used for the production of droplets. In this process, multiple emulsions can also be used, but the most common one is a double emulsion.^108^

###  By single emulsion MF technique

It involves injecting the dispersed phase into another partially immiscible or completely immiscible liquid phase, i.e., the continuous phase via an MF device. Droplets are nipped off at the meeting of two phases junction. The most commonly used geometries are T-junction, flow focusing, and co-flowing MF devices.^108^ Highly monodisperse spherical microparticles of size ~200 µm was produced utilizing the single emulsion (O/W) MF technique. The microparticles consisted of drug crystals and an excipient of macromolecular size, with its controlled morphology and polymeric outcome.^109^

###  By double emulsion MF technique

MF can be utilized to form W/O/W emulsions or O/W/O emulsions. The size of inner or outer droplets, the number of inner droplets, and the thickness of the middle layer can be adjusted by the adjustment of MF geometry and relative flow rates.^108^ This technology was used in the preparation of loading of antacid drugs in microcapsules cross-linked chitosan. The double emulsion MF technique was employed to make hollow microcapsules, which were followed by photopolymerization of polymers.^110^ The application of MF-produced multiple emulsion droplets can be utilized to make microparticles, microgels, and microcapsules.

###  Electrochemical droplet-based MF technique

The combination of the electrochemical detection framework along with MF innovation is an appealing technique for electroanalysis of samples in a small volume (μL or nL). They are highly adaptable, easy to execute with low creation costs and quick analysis.^111^ Chip-based carbon paste electrodes were fabricated for the determination of ascorbic acid (AA) and dopamine (DA) in intravenous drugs using droplet-based MF combined with chronoamperometric detection. Relative standard deviations were found to be less than 5% for intra-day and inter-day measurements and hence was proven to be highly reproducible for the analysis of DA and AA.^112^ Gu et al^113^ developed an MF microfabricated chip utilizing platinum (Pt) NPs as a proficient biosensing stage for the detection of glucose in human blood serum tests. The MF chip was developed dependent on PDMS utilizing delicate lithography. The subsequent microfluidic sensor showed a linear response of up to 43.5 mM glucose with replicability of 2.65% RSD.

## Future in research and market

###  Nanomedicine manufacturing scale-up 

NPs formulation employs multiple steps like centrifugation, extrusion, homogenization, etc. which are tedious and time-consuming. Such processes can be manageable at small scales but at a large scale, it can be an issue to achieve reproducible manufacturing of NPs at a reasonable cost. Further, the product integrity and property need to be maintained throughout the manufacturing process, which requires an appropriate understanding of the process, various control assessments, and experienced staff. Automation and continuous flow in the system are required to overcome such problems, which can be achieved via MF. MF systems can be employed for manufacturing various NP formulations and liposomes.^114^

There are some MF-based devices, which are commercially available for NP preparation using the parallelization process. One such device is the Nano assembly by Precision NanoSystems that allows NP and liposomes preparation using parallelized MF units. It consists of pumps; replaceable mixing cartridges, syringes, and an interface that controls the setup. They are a large-scale system that operates under current good manufacturing practices conditions.^115^

###  On-demand production of pharmaceuticals

On-demand production of nanomedicines can be appropriate for personalized drug products, multiple drug products with multiple formulations and low stability products meant for immediate consumption along with many more other reasons.^116^ With the recent advances in continuous manufacturing and flow chemistry, it is a possibility that end-to-end on-demand nanomedicine production may become a reality in the upcoming time. Flow synthesis platforms can be based on MF-based production as a downstream process. Personalized medicines could be made available by utilizing on-demand production pharmaceuticals utilizing MF technique.^117^

###  Wearable MF

Wearable devices that incorporate MF elements in their processes are MF wearables. Some advantages of these devices are the precise handling of a small volume of liquids, which allows accuracy and reliability. Entrapment and storing of solutes can also be done in the case of controlled drug administration.^118^

A sweat monitoring wearable device was developed where the device could be implemented at different body locations. Measurement parameters, which were studied included, sweat loss, lactate, pH, chloride, and glucose. It was utilized to evaluate athletic skin health and performance.^119^

Owing to its numerous benefits over conventional techniques, there has been wide interest in MF in the market for its commercial values and nanotechnology, and biotechnology. Until 2020, there has been a US$ 6 billion market for MF devices.^120^ Major market share belongs to MF devices related to healthcare and life sciences such as drug discovery and drug delivery, and diagnostic MF devices, which are considered as “killer applications” of MF. This new and emerging technology also has the potential to cause a revolutionizing impact on chemical synthesis and analysis, owing to the reduced number of steps and reproducibility. Scientists from all over the world have started realizing the true potential of MF and started pursuing it, making it a multidisciplinary field.^120^

There are already some MF devices available in the market, some of them are mentioned in Table 5.^121^

**Table 5:** Marketed MF devices

**Company**	**MF device**	**Sources**
Metrigenix	Microarray devices for bioanalysis	http://www.metrigenix.com/
Microsaic Systems	Integrated mass spectrometer chip	https://www.microsaic.com/
Nanogen	NanoChipÒ Molecular Biology Workstation	https://www.nanogen.com/
Randox Laboratories	Biochips for diagnostic applications	https://www.randox.com/
Shimadzu Corporation	Protein array analysis	http://www.shimadzu-biotech.net/
Solexa	Single-molecule array nanotechnology	https://www.solexa.com
Tecan	Microfluidics platform for measuring drug-drug interactions	https://www.tecan.com/
Velocity 11	Nanoliter dispensing and handling system	http://www.velocity11.com/
Vysis	GenoSensorÔ Microarray Platform	http://www.vysis.com/
Zeptosens	Sensichip/protein microarray	http://www.zeptosens.com

## Conclusion

 MF has a vast potential for simplifying tedious in-lab procedures for processes such as drug discovery and drug manufacturing to convert them into a device as small as a chip. This can revolutionize the pharmaceutical industry by making the majority of the processes easier and faster, also requiring lesser space due to their portability. Wearable MFs are also a state-of-the-art technology, which can make personalized medicines available to patients. MF has numerous and unremitting applications in the pharmaceutical field and its true potential are slowly starting to be realized. It has various benefits, such as using small volumes of reagents, parallelization, quicker response, rapid screening, continuous flow operations, and portability. All these qualities of MF devices can lead to the production of intelligent drug delivery devices. This technology can be evolved continuously according to the rising needs in drug discovery and development trends and has caused a paradigm shift in the way pharmaceutical and biological research is performed. There are also some challenges, which need to be addressed. This technology is not yet widespread and will require many more years for its easy access to industries. In addition, these devices are not user-friendly and are complex systems, therefore highly skilled personnel are needed to operate them. Their reliability is yet to be satisfactorily proven at a higher scale, as most of the research done on MF is only lab-scale. Hence, MF technology needs to be developed further to overcome all the challenges and to display its true potential to the masses.

## Ethical Issues

 Not applicable.

## Conflict of Interest

 None declared.
